# Linearity Enhancement Techniques for PGA Design

**DOI:** 10.3390/mi14020356

**Published:** 2023-01-31

**Authors:** Yujun Wang, Yi Wang, Lixi Wan, Zhi Jin

**Affiliations:** 1 Institute of Microelectronics of Chinese Academy of Sciences, Beijing 100029, China; 2 University of Chinese Academy of Sciences, Beijing 100029, China; 3 Chengdu Tiger Microelectronics Research Institute Co., Ltd, Chengdu 610000, China; 4 School of Integrated Circuits, Tsinghua University, Beijing 611731, China

**Keywords:** PGA, class AB, pre-charge, linearity

## Abstract

This paper presents some techniques to improve the linearity of traditional resistive feedback PGAs. By utilizing the switched op-amp in the PGA, the MOS switches in the feedback resistor array can be eliminated and thus the PGA’s linearity can be improved. The PGA’s linearity is further improved with an additional capacitor, which is used for pre-charging the sampling capacitor to strengthen its capability to drive the sampling capacitor without any extra power consumption. The pre-charge technique is especially suitable for the case where the PGA drives a large sampling capacitance. Implemented in SMIC 0.18 um CMOS technology, the proposed PGA can achieve a gain of 0.5 or 1 and consumes 4.68 mW at a single 5 V supply with the switched output stage enabled. When driving a 20 pF sampling capacitor at a sampling frequency of 200 kHz, the simulation results show that the proposed PGA can give a 9 dBc improvement in SFDR of the sampled signal compared to the traditional PGA design and the SFDR can reach up to 114 dBc.

## 1. Introduction

**Figure 1:**
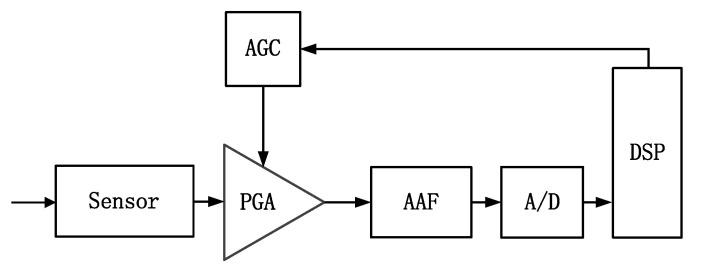
Block diagram of a typical analog front-end.

The programmable-gain amplifier (PGA) is one of the fundamental building blocks in most analog front-ends and is widely used in wireless communication [1,2,3,4], bioelectronic signal processing [5] and data acquisition systems [6,7,8], whose main function is to provide a relatively constant input level to optimize the dynamic range of these systems. Figure 1 shows the block diagram of a typical analog front-end, which is composed of a sensor, an anti-aliasing filter, an AD converter, a digital signal processor and an automatic gain control loop in addition to a PGA. Additionally, the gain control of the PGA is performed by a DSP through the automatic gain control loop.

There exist mainly two topologies for the implementation of the PGA: the negative feedback closed-loop architecture [2] and the open-loop architectures [9,10]. The gain of the closed-loop PGA is dependent on the ratio of the feedback resistance to the input resistance and is insensitive to PVT variations. However, implementing such a PGA demands an op-amp with an extremely high gain and sometimes also requires a wide bandwidth which means that a power hungry op-amp is needed. On the other hand, open-loop PGAs implement the programmable gain by using the variable input transconductance [9] or output load [10], and the requirements on op-amps are quite relaxed. However, open-loop PGAs suffer a lot from PVT variations. Additionally, the latest research about the PGA has been mainly focused on its applications in wireless communication systems [11,12,13,14,15]. Wide bandwidth and wide gain tuning range are usually required for PGAs utilized in these systems, while the linearity may be limited. For example, the THD performance in reference [11] is only about −50 dB and is not suitable for some applications requiring high linearity. Traditional resistive feedback PGAs are still widely employed in the cases [8,9,16,17]. However, the linearity of the traditional closed-loop resistive feedback PGAs is limited by the nonidealities of the MOS switches utilized in the feedback resistor array network. Meanwhile, another issue in traditional closed-loop PGAs is their lack of capability to drive sampling circuits. In order to obtain a high precision sample to the input signal, the traditional solution is to give more of a power consumption budget to the PGA or spend more time on the sampling phase, which means that a trade-off between power consumption and speed has to be made. It is really a challenging job to design a high linear PGA without experiencing losses in power efficiency and speed.

In this paper, a novel PGA architecture is proposed. A switched op-amp is introduced into PGA design, and the MOS switches used in the feedback resistor array are eliminated. In doing so, the linearity of the PGA can be enhanced. A capacitor used to pre-charge the sampling capacitor is also employed in the proposed PGA to strengthen its capability to drive sampling circuits, and thus the linearity is further improved.

The remainder of this paper is organized as follows. Section 2 gives a detailed analysis of traditional closed-loop PGAs and points out the nonideal factors that limit their linearity. Both the switched op-amp and pre-charge techniques used in the proposed PGA are introduced in Section 3. Simulation results are given in Section 4 and, finally, some conclusions are given in Section 5.

## 2. Analysis of the Traditional Closed-Loop PGA

**Figure 2:**
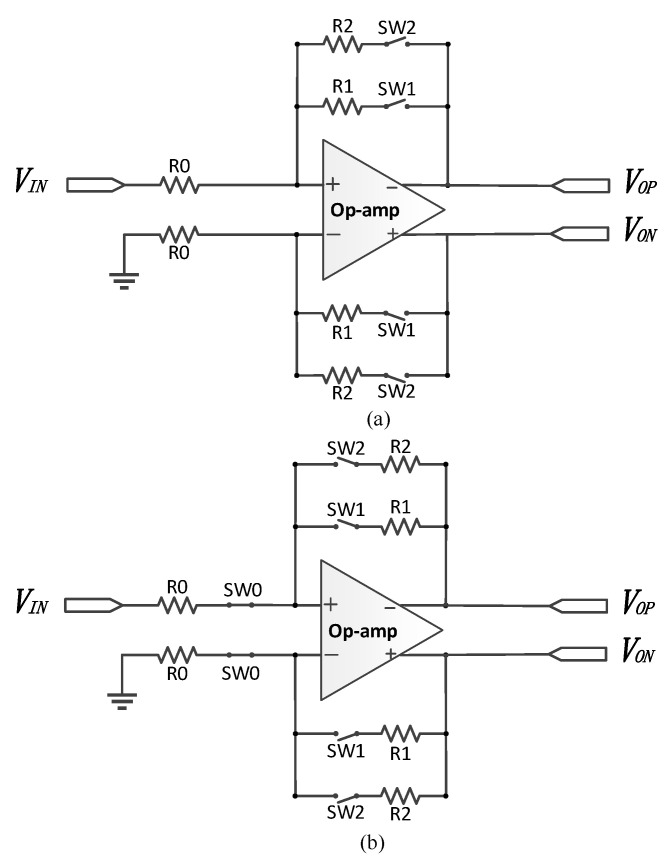
PGA architectures with (**a**) control switches at the output of the op-amp and (**b**) control switches at the input of the op-amp.

The traditional closed-loop PGA architectures are shown in Figure 2. As can be seen, the control switches can be placed at the input or output ends of the op-amp, and the PGAs shown below also perform a single to differential conversion. To simplify, the feedback resistor array network here only consists of two switched resistors. By adding more switched resistors, the gain range of the PGA can be extended. Assuming the gain of the op-amp is infinite, and only SW1 is closed, the closed-loop gain of the PGA in Figure 2a is expressed as:

(1) $$Gain1=\frac{R_{1}}{R_{0}}$$

The gain control is realized by different combinations of feedback resistors; tuning the switches in series with the resistors in the feedback resistor array will achieve the programmable gain. However, in Equation (1), the on-resistance of the MOS switches has been neglected. Indeed, the gain in (1) should be rewritten as

(2) $$Gain1=\frac{R_{1}+ron1}{R_{0}}$$

where *ron*1 is the on-resistance of SW1. This will not bring any nonlinearity to the gain but only a constant gain shift if ron1 holds a constant value. Such a switch can be implemented with an NMOS transistor; then, the on-resistance of SW1 can be expressed as

(3) $$ron1=\frac{1}{\mu_{n}C_{ox}\frac{W}{L}\left(V_{DD}-V_{TH}-V_{OUT}\right)}$$

where *μ_n_* is the carrier mobility, *Cox* is the gate oxide capacitance per unit area, *V_TH_* is the threshold voltage and *V_OUT_* is the output voltage of the PGA, where all of which are constant values except for *V_OUT_*. *V_OUT_* is dependent on the input of the PGA; thus, *ron*1 is related to the input of the PGA and nonlinearity is introduced in Equation (2). Even though SW1 can be implemented through a transmission gate, the on-resistance of SW1 is still slightly related to the input of the PGA, which still results in degradation in the linearity.

By placing the switches at the input end of the op-amp and proper sizing arrangements, the linearity of the PGA can be kept free from the varying on-resistance of the switches as shown in Figure 2b.

In Figure 2b, SW0 is an always-on dummy switch and the switches are sized according to Equation (4).

(4) $${\left(\frac{W}{L}\right)}_{0}:{\left(\frac{W}{L}\right)}_{1}:{\left(\frac{W}{L}\right)}_{2}=\frac{1}{R_{0}}:\frac{1}{R_{1}}:\frac{1}{R_{2}}$$

Then, Equation (5) can be derived, where *ron*0, *ron*1 and *ron*2 are the on-resistances of SW0–SW2, respectively.

(5) $$\frac{R_{0}}{ron0}=\frac{R_{1}}{ron1}=\frac{R_{2}}{ron2}$$

It should be noted that Equation (5) is established, unrelated to the input of the PGA. Hence, the gain with SW1 being closed can only be expressed as

(6) $$Gain1=\frac{R_{1}+ron1}{R_{0}+ron0}=\frac{R_{1}+\frac{R_{1}}{R_{0}}ron0}{R_{0}+ron0}=\frac{R_{1}}{R_{0}}$$

where *ron*0–*ron*1 are the on-resistances of SW0–SW1, as previously mentioned. As can be seen, the nonlinear on-resistances of SW0 and SW1 are canceled out. The gain is now precise and highly linear compared to Equation (2). However, the switches have introduced nonlinear parasitic capacitances at the input nodes of the op-amp, which will lead to the degradation in linearity at high-frequency inputs.

**Figure 3:**
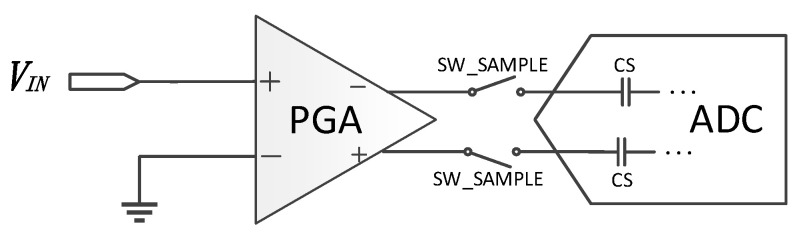
Typical block diagram of a data acquisition system.

There comes another problem when the PGA is driving a large sampling capacitance in some particular applications [16,17], as shown in Figure 3.

At the beginning of the sampling phase, the sampling capacitor CS will be connected to the output node of the PGA and acts as a load to the PGA. The sudden load of the sampling capacitor CS to the PGA will cause the slewing of the op-amp inside the PGA especially when the initial PGA output voltage differs a lot from the last sampled voltage on the sampling capacitor, which will reduce the settling speed of the output voltage. In other words, the settling speed is dependent on the previously sampled signal, which will also cause degradation in linearity with a fixed sampling duration. Whether giving more power consumption budget to the PGA to speed up the settling of the sampling signal or prolonging the sampling duration, neither are a perfect solution.

From the above analyses, the linearity of traditional closed-loop PGAs is limited by the nonideality of MOS switches in the feedback resistor array network and the sudden load of sampling capacitor CS to the PGA during the sampling duration.

## 3. The Proposed PGA Architecture

In this section, a novel PGA architecture is proposed. Firstly, the op-amp in the traditional closed-loop PGA is replaced by a switched op-amp (SC-OPA), and the MOS switches in the feedback loop are eliminated. As a result, the gain linearity is improved. Furthermore, an additional capacitor used to pre-charge the sampling capacitor is also employed to speed up the settling of sampling signal, which further improves the linearity of the PGA. A detailed description about these techniques will be given below.

### 3.1. PGA Design Based on a Switched Op-Amp

**Figure 4:**
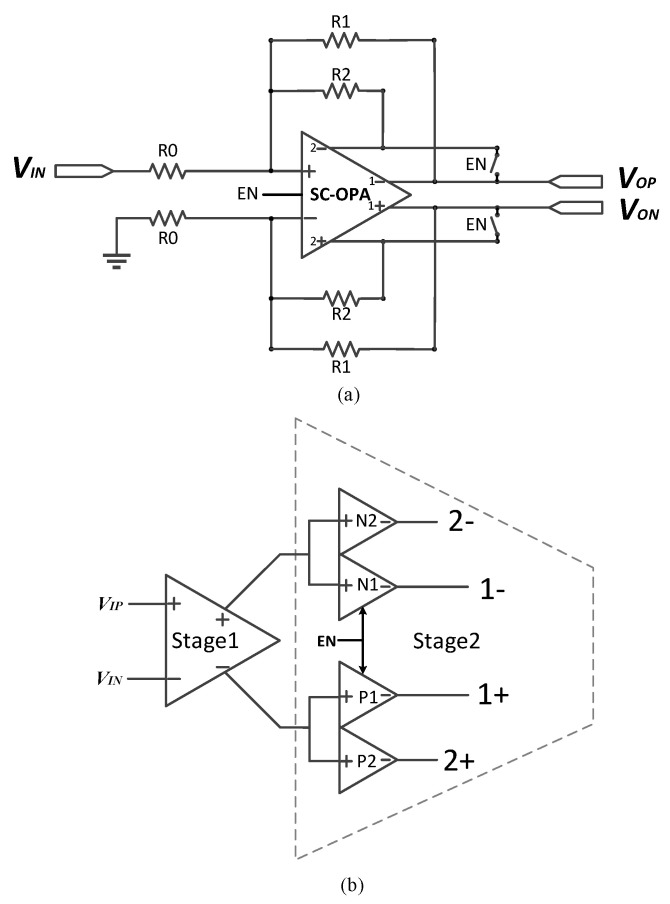
Block diagram of (**a**) the proposed PGA and (**b**) SC-OPA.

Figure 4a displays the block diagram of the proposed PGA architecture and Figure 4b shows the block diagram of the switched op-amp. As can be seen, the switched op-amp has two output stages that share the same input stage. Additionally, each output stage corresponds to a feedback resistor array. When the EN signal is enabled, both *R*_1_ and *R*_2_ participate in the signal amplification process, and the gain is expressed as follows:

(7) $$Gain1=\frac{R_{1}∥R_{2}}{R_{0}}$$

Additionally, when the second output stage of the second stage is disabled, only *R*_1_ participates in the signal amplification process; thus, the gain now turns into

(8) $$Gain2=\frac{R_{1}}{R_{0}}$$

Because the MOS switches presented in the feedback loop of the traditional PGA now disappear, a more linear gain can be obtained compared to traditional closed-loop PGAs.

Different from the conventional design, the resistor array is directly connected to the output stage without any serial MOS switches. The control of the feedback resistor array is realized by controlling the corresponding output stage; when we do not want some resistor to participate in the signal amplification process, we can simply disable the corresponding output stage and vice versa. In such a manner, the programmable gain can be achieved. Additionally, the gain range can be extended by adding more switched output stages and feedback resistor arrays.

### 3.2. The Proposed Pre-Charge Technique in the PGA

**Figure 5:**
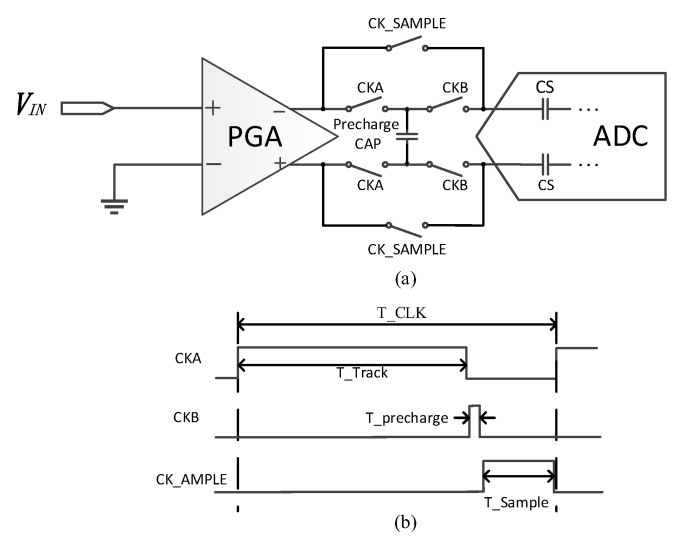
(**a**) Block diagram of the pre-charge technique used in the PGA. (**b**) Timing diagram of the pre-charge technique.

Figure 5 shows the proposed pre-charge technique and the timing diagram, respectively. A third phase is added between the tracking phase and sampling phase, which is called the pre-charge phase. Additionally, an additional capacitor is also added to the output of the PGA. During the tracking phase, the switches between the PGA and this additional capacitor are closed; hence, the capacitor is charged by the PGA in this phase. This charge operation ends at the end of the tracking phase; then, the switches between this additional capacitor and the sampling capacitors are closed during the pre-charge phase to pre-charge the sampling capacitors and thus they perform a coarse sample to the input signal. Finally, during the sampling phase, the sampling capacitors are connected to the PGA to realize a fine sample to the input signal. In this way, two-step sampling to input signal is achieved. The slewing effect of the op-amp has been weakened thanks to the pre-charge to the sampling capacitors and thus this speeds up the settling of the sampling signal. In other words, the linearity of the sampled signal will be improved with the same sampling duration.

The speed of the system is not affected by the additional pre-charge phase, because the duration of the pre-charge phase can be taken from the original sampling phase duration and is short. In addition, the linearity of the PGA can be improved without any extra power consumption.

### 3.3. The Circuit Implementation

**Figure 6:**
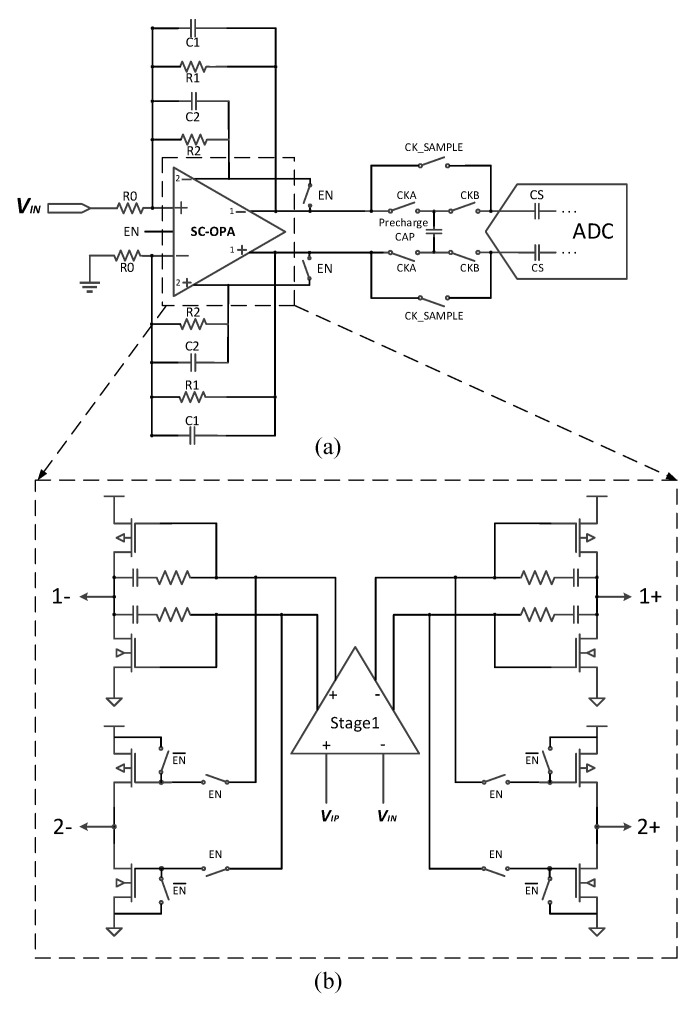
(**a**) The proposed PGA design. (**b**) Schematic of the SC-OPA.

Figure 6 shows the final block diagram of the proposed PGA and the circuit schematic of the switched op-amp. Each resistance of the feedback resistor array is set to be identical to the input resistance of the PGA (*R*_0_ = *R*_1_ = *R*_2_); thus, the proposed PGA can realize a gain of 0.5 or 1. The feedback resistor along with the parallel capacitor form a first-order anti-aliasing filter.

The switched op-amp is a two-stage amp consisting of a shared input stage and two class ab output stages. In Figure 6b, the upper output stage is the main output stage and is kept always-on, while below is the auxiliary switched output stage. By placing some switches at the gates of the auxiliary output stage, a switched amp is realized. The switched amp can be enabled with its input connected to the input stage or disabled with its input connected to the VDD or GND. This way, we can determine whether the corresponding feedback resistor participates in the signal amplification or not, and gain control is realized in this way. Additionally, note that in Figure 6b the miller compensation is only realized between the first stage and the main output stage to save the chip area.

Generally speaking, the additional capacitance should be set as large as possible to perform an efficacy pre-charge to the sampling capacitor. Yet, a too-large capacitance may introduce a stability issue in the feedback loop. So, the ratio of the additional capacitance to the sampling capacitance is set to two to guarantee the stability of the feedback loop without too much loss in pre-charge efficacy.

## 4. Simulation Results

**Figure 7:**
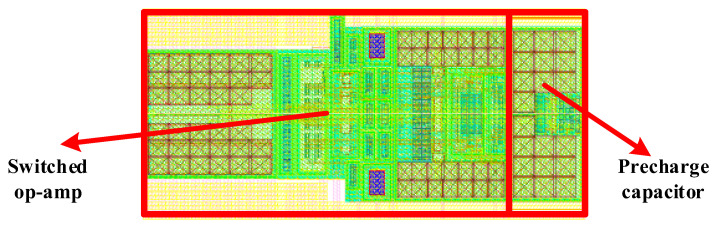
Layout of the proposed PGA.

The prototype PGA is implemented in SMIC 180 nm technology using the proposed techniques and the layout is shown in Figure 7. The proposed PGA occupies a die area of 0.17 mm^2^ including a switched op-amp and a pre-charge capacitor. The performance of the switched op-amp is summarized in Table 1. Operating at a 5 V supply voltage, the power consumption at room temperature is 4.68 mW with the switched output stage enabled.

**Figure 8:**
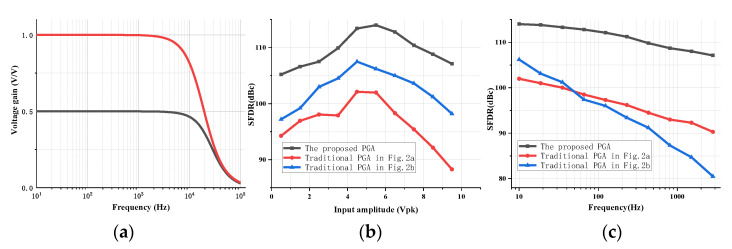
(**a**) The closed-loop gain verse frequency. (**b**) The simulated SFDR versus input amplitude. (**c**) The simulated SFDR versus frequency.

The measured voltage gain versus frequency is shown in Figure 8a. The proposed PGA can realize a gain of 0.5 or 1 by controlling the switched output stage. To verify the linearity enhancement effects of the proposed techniques, the linearities of the proposed PGA and traditional closed-loop PGAs are simulated. The gain of all of the PGAs is set to be 0.5 and each output of the PGAs drives a 20 pF sampling capacitance during the simulation. Figure 8b,c shows the measured SFDR versus input amplitude and frequency, respectively. The SFDR here is defined as the ratio of the signal power to the power of the largest undesired harmonic or spur.

As can be seen in Figure 8b, while the linearity of the traditional PGA with switches at the output of the op-amp suffers a lot from the nonlinear effects of MOS switches, placing these MOS switches at the inputs of the op-amp will keep the linearity free from the above nonlinear effects. However, the linearity is still limited due to its poor capability to drive large sampling capacitances. The proposed PGA can give a maximum SFDR performance improvement thanks to the proposed techniques. As depicted in Figure 8c, there is little degradation in linearity in the proposed PGA as input frequency increases while the measured SFDR with the traditional PGAs degrades a lot. There is a significant SFDR degradation in the conventional PGA with control switches at the inputs of the op-amp because of the introduced nonlinear parasitic capacitance. The SFDR is at least improved by 9 dBc with the proposed techniques and can reach up to 114 dBc.

**Figure 9:**
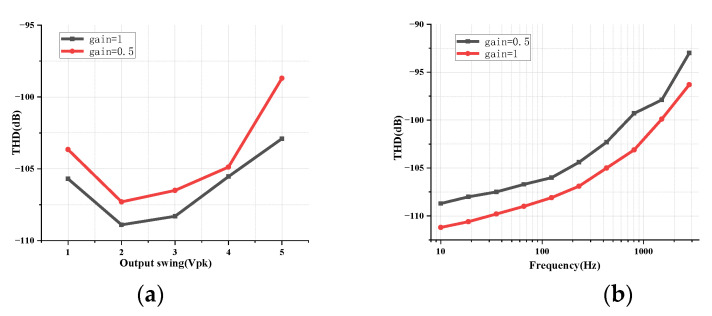
The simulated THD versus (**a**) the output swing at 1 KHz and (**b**) the frequency when the output swing = 5 Vpk.

Figure 9a shows the simulated THD versus the output swing for different gain settings. Figure 9b displays the simulated THD versus the frequency. Both pre-simulations and post-simulations on different PVT corners (tt, ss and ff) with temperatures of −40 °C, 27 °C and 85 °C are carried out. Results are summarized in Table 2. As can be seen, the proposed PGA design is robust and the linearity degrades little in post-simulations. The main performances and comparisons with some existing works are summarized in Table 3.

## 5. Conclusions

This paper presents a PGA design with high linearity performance. Firstly, the switches in the feedback loop of the traditional closed-loop PGA are eliminated by introducing a switched op-amp into the PGA design which is beneficial to the linearity of the PGA. The PGA’s driving capability is enhanced with the proposed pre-charge technique and thus this leads to a further improvement in linearity. Moreover, this would not consume any extra power. Implemented in SMIC 180 nm technology, the proposed PGA can provide a gain of 0.5 or 1 and gain error of less than 0.0013 V/V. It occupies a die area of 0.17 mm^2^ and the total power consumption at a 5 V supply is 4.68 mW with its switched output stage enabled. Compared with previously published works as displayed in Table 3, the proposed PGA has obvious advantages in terms of the THD and gain error. The THD performance is still better than −98 dB at a 5 Vpk 1 KHz sinusoidal input. Thus, it can be applied to systems where high linearity is required.

## Figures and Tables

**Table 1:** Performance of the switched op-amp with its switched output stage enabled and disabled.

	Open-Loop Gain (dB)	GBW (MHz)	Phase Margin (deg)	Power (mW)
Enabled	101.1	11.2	91	4.68
Disabled	100.9	10.94	87.1	4.39

**Table 2:** Summarization of simulation results.

Input amplitude = 5 Vpk at 1 KHz gain = 1		Typical	Min	Max
Pre-simulation	SFDR (dB)	105.2	91.7	108.6
	THD (dB)	−103.2	−105.8	−87.2
Post-simulation	SFDR (dB)	104.7	92.2	108.8
	THD (dB)	−102.9	−105.1	−88.3
Input amplitude = 10 Vpk at 1 KHz gain = 0.5		Typical	Min	Max
Pre-simulation	SFDR (dB)	103.2	101	108.7
	THD (dB)	−98.8	−105.8	−95.8
Post-simulation	SFDR (dB)	103.8	101	107.3
	THD (dB)	−98.4	−105.8	−96.9

**Table 3:** Performance summary and comparison.

Reference	[11]	[13]	[14]	[15]	This Work
Technology (nm)	180	180	180	180	180
Supply (V)	5	1.8	1.8	1.2	5
Bandwidth (MHz)	30	10–25	14	11–78	0.02
Gain range (dB)	0–14	−12–24	3.39–43.79	19.5–42.5	0.5/1 V/V
Gain error (dB)	<0.5	N/A	0.07	N/A	0.0013 V/V
THD (dB)	−50.5	<−56 ^a^	N/A	−40.7 ^a^	−98.4
Power (mW)	1.044	3.6	7.02	0.1968	4.68
Area (mm^2^)	0.052	0.32	0.283	0.0007	0.17

^a^ HD3.
